# Annotations

**Published:** 1892-04-02

**Authors:** 


### Annotations.

**Our New Departure.** — With the present number, The Hospital enters upon a new phase of its life. The paper has now been in existence for more than a twentieth part of a century. A newspaper that has survived so long and that has grown stronger with every year of its history has fairly established a claim to permanence and to such development as the necessities of its readers and the nature of its objects may properly demand. There has long been a need for the enlargement of the paper. Much matter of importance and interest to various classes of readers has constantly had to be excluded for want of space. The proper work of th e paper has grown beyond all anticipation. In early days friendly critics used to ask how we proposed to continue to fill our pages with interesting matter on such a comparatively limited range of subjects as hospitals seem to include? We confess that the question sometimes wakened uneasy echoes in our own minds; but experience has shown that neither readers nor ourselves measured quite accurately the range of our work, or saw with true vision the large numbers of persons of all shades of opinion who were interested in hospital questions, or gauged aright the depth and intensity of their regard for the work of medical charity. We all know better now. Whatever may be the feelings of our constituents towards The Hospital and its conductors, we ourselves have but one feeling towards them. By their loyal, constant, and increasing appreciation the paper has been made successful beyond all precedent and even beyond hope. On this point we feel that to say the least is to say the most. We beg all classes of readers to receive our heartfelt thanks. It is our purpose, from the present time, to make the paper more truly special and institutional; though not, therefore, narrower in the range of its subjects or more limited in the variety of its interests. In the old days all roads led to Rome; and in these times every father and mother, every husband and wife, every brother and sister, and indeed every human being, great or small, has some kind of interest which draws and attaches him to the sick and the suffering, and to the methods that are adopted for their relief. Moreover, medicine, including as it does physiology, human and animal, has become one of the most variously interesting and progressive of all the sciences. The time is close at hand when it will be considered as much a mark of defective education to be ignorant of physiological science in its widest sense as it now is to be ignorant of history, politics, or literature. One of the chief aims of The Hospital in future will be to make known to the public, as well as to the medical profession and to hospital constituents, the larger issues of medical and physiological science. During the last half century medicine has awakened out of an almost death-like sleep. She has come forth from the grave, and all the world has opened curious eyes upon her to see what manner of spirit she is of. There is, therefore, a peculiar necessity for a paper like The Hospital to interpret to the larger world the benevolent, the philanthropic, the scientific, and the broadly humane spirit of the medicine of modern times. From the first it has been our consistent policy to stand by institutions which diffuse the large and indispensable benefits of medical science directly among the poor, and indirectly among all sorts and conditions of men. This line of policy was taken up, not from ignorance, but from knowledge, not from prejudice, but from an assured conviction that medical charities, and medical charities voluntarily conducted, are as indispensable to the well-being of civilised communities as Government and education—we had almost said—as sunshine and fresh air. Just as we have stood firmly by these institutions in the past, so we propose to stand even more firmly by them in the future. Our widening experience has shown us that whilst their faults are infinitesimal, their virtues and benefits are universal. We enlarge our borders with pleasure, and not without a little modest pride. In future we shall be able to offer to readers and writers, and to institutions and their governors and officials, ample space for the discussion of all kinds of theoretical and practical questions bearing upon the larger medicine, and upon the greater and smaller charities by which it is represented to the world. This expansion will, we hope and believe, be both interesting and helpful to all classes of our readers; and we venture to ask them for a cordial renewal of that sympathy and confidence which have always been so steadily given to the paper, from the first day of its history until now.

**Some Good Women.** — Many readers of the *Daily Graphic* must have been struck by the pleasing pictorial presentation of the opening of the new wing of the central shelter of the National Society for the Prevention of Cruelty to Children by Lady Salisbury. The picture shows a couple of small rescued girls, one of them a bright and pretty little creature, presenting the wife of the Premier with a bouquet. Lady Salisbury is in the act of receiving the gift with a truly motherly smile, whilst Lady Iddesleigh, the Countess of Wharncliffe, and several other beautiful and aristocratic women look on admiringly. One cannot help feeling that in such circumstances as these women are seen at their very best. No sensible man can have any philosophical objection to lady doctors, lady teachers, literary women, and the like. But when all this is admitted one still feels that women please us best, and most compel our love and loyalty when they are giving themselves up to the softening of the asperities of life, to the motherly protection of children, their own and other people's, and to the making of peaceful and restful homes for their fathers, their brothers, and their husbands, who must perforce engage in hand to hand fights with destiny. The writer confesses to a sneaking sort of sympathy with Mr. Walter Besant, who wants women to have soft cushions to sit upon, and pathways smoothed for their feet. So tough a fighter as St. Paul seems to have been inclined to Mr. Besant's view when he counselled husbands to "give honour to their wives as to the weaker vessels." Mr. Benjamin Waugh has proved himself a shrewd diplomatist in engaging the hearts of so many noble British mothers and sisters on behalf of the much injured children of his truly worthy Society. Their influence will, no doubt, help him to soon clear off his debt of £3,000, and to establish the three hundred centres that are required instead of the ninety which now constitute the limit of the Society's operations.

**The "Year Book of Science."** — Some time ago Messrs. Cassell and Co. promised a "Year Book of Science," and now that the book is issued one cannot help wondering why it was never issued before. Professor T. G. Bonney, F.R.S., is the editor, and the contributors, although not numerous, are competent and pretty widely representative. Taking at random an article on a physiological subject, one finds something that has the merit of being comparatively new, and decidedly instructive. The article deals with the specific gravity of the blood. Now the specific gravity of the blo od is no doubt ordinarily an approximate measure of its richness, and therefore of its nutrient and sustaining properties. Among other things the writer of the article informs us that the specific gravity of men's blood is higher than that of women, a statement which we can readily believe. This difference does not show itself until about the age of fifteen, and that, no doubt, for reasons which will at once occur to the physiologist. In dark persons the density of the blood is higher than in those of lighter complexion. For this an ethnological reason is suggested, namely, the incomplete fusion of the races from which the present inhabitants of Britain have been derived. The writer considers that those persons among us whose blood is of more watery type have descended from Saxon and Scandinavian ancestors. Be this as it may, the suggestion is interesting, and opens out a wide field of enquiry and speculation. A poor physique is said to accompany a low specific gravity of the blood; and that also most people will believe. If this fact be taken in conjunction with the following—that regular exercise and sound sleep tend to raise the specific gravity of the blood—we have a scientific confirmation of the popularly believed, but too little-practised theory, of the health value of vigorous exercise in the open air and a restful state of mind. This is but one of some hundreds of topics which the New Year Book briefly and intelligently discusses. The volume will no doubt be extensively purchased by all who are interested in the current progress of science, and we may predict for it, not only an extensive sale but a continued improvement from year to year. It is a work which once having been begun can never again be dispensed with.

